# Supplementary material for: Chromothripsis during telomere crisis is independent of NHEJ, and consistent with a replicative origin
Source: Genome Res. 2019 May;29(5):737–49. doi: 10.1101/gr.240705.118 (PMC6499312; doi:10.1101/gr.240705.118)
Supplement: Supplemental Material [file supp_gr.240705.118_Supplemental_file_1.zip › contigs/annotated_contigs/DB116/contig.2.DB116_length_566_mean_cov_9.55830388693.docx]

**DB116_length_566_mean_cov_9.55830388693**

```
TACTGGGTAAACTCCTATTCTCTCATTTCAAATTTTTTCTATTATCACACCATATGTAAATCTATTTTGCCCTCTTCAAGGTAGATTCT
 >chr11:80570826-80571068 + E=6e-134 p=3e-03
TCTCCACTTGTGTTTCTACGGTTCGATTCAATTATTGTCCTTATCATTTTTCAGCTATTATTTGTTTAAATGCTTGTACCTTGGAAGAT

GAAATCTGTCTCTAAATCCATCTTTGTATTCCTTGAAATGAATATATATATATATATATATA|TA|AAAAACAGCAAAAAATTAAATAA
                                                               >chr11:80570127-80570453
GTAACATGTGGTCTCTAATGTGGTAAGTGATTTGATAATATTTAATACAATAAATGAAGGGATCATATTTCAACGTATTTGCCAATGAC
 - E=1e-181
ATTATCAAAATCAAGAAAAAAAAACTAAGCTAGGGATAAATACACATAAAACTCAGCATAGGGGATAAAGGTAGAAAGTATCAAGTGTT

TAATTTCAAAAAGAGATAAACCCCTGCTTTCGCATCAGGCTATAAGAAAAGTTTTGAGTATTATATTTGACCCAAAGCATAAACAGGAT

TAACTATGTTGATGCTAGTATTTGTTTTAAATTA
```
